# Trends in cannabis-related emergency department visits and hospitalizations among children aged 0–11 years in Canada from 2015 to 2021: spotlight on cannabis edibles

**DOI:** 10.1186/s12889-023-16987-9

**Published:** 2023-10-23

**Authors:** Melanie Varin, Andre Champagne, Jeyasakthi Venugopal, Le Li, Steven R McFaull, Wendy Thompson, Stephanie Toigo, Eva Graham, Anne-Marie Lowe

**Affiliations:** Public Health Agency of Canada, Ottawa, ON Canada

## Abstract

**Background:**

Cannabis poisonings among children are of public health concern. Existing evidence from the US and from four provinces in Canada (Quebec, Ontario, Alberta, British Columbia) indicate an increase in pediatric cannabis-related poisonings since the legalization of cannabis. This study evaluates trends in cannabis-related poisoning pediatric emergency department (ED) visits and hospitalizations in Canada and addresses a gap in literature by describing trends and context around cannabis edible-related poisoning cases using data from a Canadian sentinel surveillance system.

**Methods:**

Mixed-methods using data from two administrative data sources and one injury/poisoning sentinel surveillance system to estimate age-specific rates of cannabis-related poisonings ED visits (Ontario and Alberta), edible-related events (sentinel surveillance Canada), and hospitalizations (Canada with the exception of Quebec) among children between the ages of 0 to 11 from 2015/2016 to 2021. Annual absolute changes were calculated to quantify the magnitude of change between each age-specific rate. Joinpoint regression was used for trend analysis. A thematic analysis was completed to gain a better understanding of cannabis edible-related poisoning cases in the ED.

**Results:**

The pediatric age-specific rates for cannabis-related poisoning ED visits (average annual percent change (AAPC) Ontario: 98.2%, 95% CI: 79.1, 119.2; AAPC Alberta: 57.4%, 95% CI: 36.7, 81.2), hospitalizations (AAPC: 63.4%, 95% CI: 42.0, 87.9) and cannabis edible-related events (AAPC: 122.8%, 95% CI: 64.0, 202.6) increased significantly from 2015 to 2021. Almost half of all pediatric edible-related events involved gummy edible products (48.8%, n = 143). Based on the thematic analysis, 88% cannabis edible-related events were attributed to inadvertent ingestion due to access to such products or lack of safe storage practices.

**Conclusion:**

Age-specific rates of cannabis-related poisoning ED visits (Ontario and Alberta) and hospitalizations (Canada with the exception of Quebec) have increased since cannabis legalization, with the largest increase in rates occurring from 2019 to 2020. A similar increase in the rate of cannabis edible-related cases from sentinel surveillance data underscores the importance of monitoring this outcome. Public health messaging and national public health promotion strategies targeted towards raising awareness on the risks associated with consuming illegal cannabis and safe storage of cannabis could help mitigate cannabis poisonings among children.

## Introduction

On October 17, 2018, the *Cannabis Act* [1] came into force, legalizing and regulating dried cannabis, cannabis oil, fresh cannabis, cannabis seeds and cannabis plants for non-medical purposes. This was followed by an amendment to the *Cannabis Regulations* [2] in October 2019 that provided access to edible cannabis products, cannabis extracts, and cannabis topicals. These other products only became commercially available to the Canadian public in late December 2019 with increasing availability into early 2020 [1, 2]. While federal legislation has only legalized access to cannabis for non-medical purposes for adults 18 years of age and older, there is general concern that having cannabis in a household may provide children with access to such products and increase the risk of harms, such as poisonings following the unintentional ingestion of cannabis [3].

Cannabis poisonings among children are of public health concern as they can have significant and serious effects such as lethargy, uncoordinated movements, shallow/depressed breathing, more serious behavioural changes [4], seizures [4], and can even lead to life-threatening situations such as coma or effects on respiration requiring the use of a ventilator [5]. Evidence from the US suggests that cannabis legalization may have led to an increase in cannabis poisonings among children [6–10]. Based on nationally representative US poison centre data from 2017 to 2019, there was a cumulative total of 4,172 calls related to cannabis exposure among children between the ages of 0 and 9 [8]. The prevalence of those calls increased annually from 2017 (n = 887) to 2019 (n = 1,963) [8]. Moreover, from 2017 to 2019, the proportion of calls related to pediatric edible exposure cases was higher in states where cannabis was legal among adults compared to states where it was not legal [8].

Similar observations have also recently been reported in two Canadian provinces [11, 12]. In Alberta, the incidence rate ratio (IRR) for unintentional cannabis poisoning emergency department (ED) visits among children aged 0–11 years was significantly higher after non-medical cannabis was legalized (October 17, 2018), compared to before (IRR: 1.77, 95% CI: 1.42, 2.20) [11]. Similarly in Ontario, cannabis exposure ED visits among children aged 0–9 years increased significantly in the period after commercial cannabis edibles were legalized compared to the pre-legalization period (IRR = 2.23, 95% CI: 1.17, 4.27) [12]. The authors of that study suggested that the commercial availability of legal edible cannabis products influenced the frequency and severity of ED visits [12]. Overall, these studies demonstrate an increase in visits to the ED for cannabis poisonings among children in two Canadian provinces since legalization [11, 12].

While there are no available Canadian data examining ED visits involving exposure to cannabis edibles specifically in the period after legalization, findings from a sentinel surveillance system of 19 selected emergency departments across Canada prior to legalization of edibles (April 2011-August 2019) demonstrated a small but increasing number of incidents involving edibles [13]. Among children less than 10 years of age, 35 cases of unintentional cannabis ingestion were captured, highlighting the need for safe storage of cannabis products and child-resistant packaging [13]. A recent Canadian study depicted an association between edible cannabis legalization and increases in unintentional cannabis poisoning hospitalizations among children between the ages of 0 and 9 in three Canadian provinces [14]. The rate per 100,000 person-years was substantially higher in the three provinces where commercial cannabis edibles are permitted (Ontario, Alberta and British Columbia [IRR 7.49, 95% CI 5.92,9.48]), which is in contrast to Quebec where cannabis edible products were prohibited during the study period (IRR 3.04, 95% CI 1.92,4.81) [14].

Taken together, evidence from the US [6–10] and from four provinces (Quebec, Ontario, Alberta and British Columbia) in Canada [11, 12, 14] indicate an increase in pediatric cannabis-related poisonings since legalization of cannabis. However, no study has reported national rates of pediatric cannabis-related poisoning hospitalizations. Moreover, prior analyses of trends in cannabis-related pediatric ED visits did not specifically identify cannabis edibles as the cause of the incident. The goal of this study was to leverage three data sources (two that are national) to better understand and capture the burden of pediatric cannabis poisonings across Canada. As such, the objectives of this study were three-fold:

1) Estimate the rates of pediatric **cannabis-**related **poisoning ED visits** from 2015 to 2021 for two Canadian provinces (Ontario and Alberta);

2) Describe ED trends and context around cannabis **edible**-related **poisoning cases** (narrative description of event) among children aged 0–11 years from 2015 to 2021 using data from a Canadian sentinel surveillance system.

3) Estimate the rates of pediatric **cannabis**-related **poisoning hospitalizations **in Canada (with the exception of Quebec) from 2015 to 2021;

## Methods

### Study design

This was a mixed-methods study design that used data from two administrative data sources and one injury/poisoning sentinel surveillance system to examine three outcomes among children between the ages of 0 to 11 years. Data from the two administrative data sources were from January 1st, 2015 to December 31st, 2021, whereas data from the sentinel surveillance system were from January 1st, 2016 to December 31st, 2021. While the sentinel surveillance system has data available in 2015, cannabis records only appeared in 2016. Both time periods cover pre- and post-legalization of cannabis (October 2018) and edible cannabis (October 2019).

### Data sources

Three data sources were used:

1. The National Ambulatory Care Reporting System (NACRS) was used to report on the cannabis-related ED visits for two provinces. ED data were retrieved from the Canadian Institute for Health Information (CIHI).

- The following closed year NACRS data sets were used: 2014/2015, 2015/2016, 2016/2017, 2017/2018, 2018/2019, 2019/2020, 2020/2021, 2021/2022.
- Geographic coverage: Although NACRS is a national database, data coverage varies by jurisdiction. The number of reporting facilities and levels of submission by jurisdiction have remained broadly consistent since 2019. Ontario, Alberta and Yukon are the only jurisdictions with complete coverage in NACRS. Due to low sample sizes, age-specific rates for Yukon were suppressed and are not included in the present study. Only cannabis-poisoning ED visit rates for two out of the three (Ontario and Alberta) are presented in this study.

2. The Canadian Hospitals Injury Reporting and Prevention Program (CHIRPP) was used to provide additional context to trends in cannabis edible-related poisoning ED visits. Data from this sentinel surveillance system were extracted from 11 pediatric hospitals and 9 general hospitals in Canada. Though it is not nationally representative, the data capture detailed descriptions of the cases presented at ED through free-text fields that are not available in DAD and NACRS. During their ED visit to a CHIRPP hospital, the patient or accompanying caregiver completed a questionnaire about the pre-event circumstances, including their narrative descriptions (i.e., “what went wrong”) [14]. Clinical details of the event were later added to the patient’s file by hospital staff, entered into the secure, web-based database by CHIRPP site coordinators and extracted by PHAC data coders [15].
3. The Discharge Abstract Database (DAD) was used to report on the cannabis-related hospitalizations. Hospitalization data were retrieved from CIHI and is representative for all Canadian provinces and territories (excluding Quebec).

- The following closed year DAD data sets were used: 2014/2015, 2015/2016, 2016/2017, 2017/2018, 2018/2019, 2019/2020, 2020/2021, 2021/2022.
- Geographic coverage: DAD data is available for all of Canada excluding Quebec.

### Outcomes

Cannabis-related poisoning ED visits and hospitalizations were classified using the *International Statistical Classification of Diseases and Related Health Problems, Tenth Revision, Canada (ICD-10-CA)*, with the code T40.7, which is used to describe poisoning by, adverse effect of and underdosing of cannabis (derivatives). Any hospitalization or ED visit with T40.7 in one of the diagnostic fields were included in the analyses.

Cannabis edible-related poisoning cases were defined as “any food or beverage product containing cannabis” and were identified by a variety of bilingual (French and English) keyword search terms in relevant free-text injury description variables in the CHIRPP database (based on 505,778 CHIRPP records extracted on July 18, 2022). The keywords included to identify cannabis-related events were: POT, SHATTER, THC, MARIJ, JOINT, MARYJ’, ‘MARIHU’, ‘MJ ‘, ‘MARYHU’, ‘CANABI’, HASH, MARYJANE, MARY JANE, BLUNT, GANGA, GANJA, SENSIMI’, DOPE, REEFER, CANNABIS, WEED, MARIJUANA. The keywords used to identify edible-related events were: Edible, Weed cookie, ‘Cooki’,‘Gumm’, Tincture, ‘Juju’, Candy, Bonbon, Brownie, Muffin, Biscuit, ‘Chocolat’, ‘Lolli’, Ingestion-related: ‘mang’, ‘ingest’, ‘ate’. Predetermined factor codes pertaining to cannabis were also used to collect all relevant records. The following factor codes were used:

- 787 F Synthetic cannabinoids, INCL K2, Spice, IZMS, Herbal Highs, Yucatan Fire, Earth, Impact, London Underground, Black Mamba.
- 7901 F Cannabis/marijuana extracts and resins, INCL hashish, hash oil, shatter, tinctures [use ONLY if the injury date is or after Oct. 17, 2018].
- 7902 F Cannabis/marijuana edibles, INCL brownies, gummies, THC-infused beverages [use ONLY if the injury date is or after Oct. 17, 2018].
- 7903 F Cannabis/marijuana products taken for medical use (must be specified), INCL cannabidiol (CBD) oil, Sativex, Epidiolex [use ONLY if the injury date is or after Oct. 17, 2018].
- 790 F Cannabis/marijuana, NFS, NEC, INCL dried leaves/flowers, joints, THC (NFS) [use ONLY if the injury date is or after Oct. 17, 2018].

### Data analysis

The CIHI closed year data was used to examine cannabis-related poisoning emergency department visits (NACRS, for Ontario and Alberta) and hospitalizations (DAD, nationally except Quebec). Analyses were conducted independently by two epidemiologists using SAS EG 7.1. To report on cannabis-related poisoning ED visits and hospitalizations among children aged 0 to 11 years, age-specific rates were calculated for each calendar year from 2015 to 2021. In this analysis, age-specific rates are crude rates, calculated for our study population (children aged 11 years and younger) for each study year (2015–2021). These rates are presented per 100,000 population. Rates were calculated using the ‘proc stdrate’ command in SAS. The numerator used was the total number of cannabis-related emergency department visits in the province of interest (Ontario and Alberta), and the total number of cannabis-related hospitalizations among children aged 0 to 11 years by study year. The denominator used was from Statistics Canada’s annual inter- or post -censal population estimates per age unit. Denominators used to calculate cannabis-related poisoning ED visits were population estimates from Ontario or Alberta. Denominators used to calculate cannabis-related poisoning hospitalizations were cumulative population estimates across all provinces and territories in Canada, excluding Quebec. Annual percent change (APC) in cannabis-related poisoning ED visits and age-specific hospitalization rates were calculated to quantify the magnitude of absolute change between each year. The Joinpoint Regression Program Version 4.6.0.0 (National Cancer Institute, USA) was used to determine whether there were any significant differences in trends for cannabis-related poisoning ED visits and hospitalizations from 2015 to 2021. The Joinpoint program calculates average annual percent change (AAPC) with 95% confidence intervals (95% CI) to identify whether there is a statistically significant change in the slope of the trend [15]. Model selection was based on permutation tests (n = 4,499) to identify the number of joinpoints [16].

To describe trends in cannabis edible-related cases in EDs, age-specific rates per 100,000 CHIRPP records were calculated. The numerator used was the total number of edible-related cases captured in CHIRPP among children aged 11 years and younger. The denominator used to calculate this rate was the total count of CHIRPP records involving children aged 11 years and younger between 2016 and 2021. Annual absolute change in age-specific rates were calculated to quantify the magnitude of change between each year. Joinpoint Regression Program was used to examine statistically significant changes in trend. A thematic analysis of open-text narrative description of cannabis edible-related cases extracted from CHIRPP was performed to gain a better understanding of cannabis edible-related poisoning cases in EDs. In terms of the thematic analysis, a three-step approach was conducted. First, the narratives of all the 293 cases were carefully reviewed until coders became familiar with the circumstances surrounding these cases using Microsoft Excel. The second step consisted of searching for patterns among the circumstances surrounding these cases and coding them accordingly. These codes were then reviewed by two analysts, who agreed upon two general themes: (i) children inadvertently ingesting edibles as such products were found to be accessible in homes or other various locations and (ii) caregiver/family member unintentionally providing edible products to children. The final step consisted of reporting a percentage breakdown of the agreed upon themes.

## Results

Age-specific rates for each of the three outcomes 1) cannabis-related poisoning ED visits, 2) cannabis-related poisoning hospitalizations, 3) edible-related cases from 2015 to 2021 are presented in Table 1. Cumulative rates are disaggregated by sex and year.

**Table 1:** Age-specific rates (per 100,000) for cannabis poisoning-related ED visits, edible-related poisoning cases, and cannabis poisoning-related hospitalizations, 2015–2021, disaggregated by sex and year

Outcome *(Data source)*	Cannabis poisoning-related ED visits *(NACRS)*		Cannabis edible-related poisoning cases *(CHIRPP)*	Cannabis poisoning-related hospitalizations *(DAD)*
**Geographical location**	*Ontario* *(n = 798)*	*Alberta* *(n = 218)*	*Canada (sentinel surveillance n =( 293)*	*Canada (with the exception of Quebec (n = 641)*
**Total (cumulative)**	6.4	4.8	58.0	2.5
**Sex** Male Female	8.0 6.8	5.2 4.1	56.0 60.5	2.5 2.5
**Year** 2015 2016 2017 2018 2019 2020 2021	0.3 0.9 1.6 2.9 5.5 14.9 18.1	0.8 1.4 1.4 4.4 6.9 8.7 9.6	N/A 4.7 4.6 23.7 43.1 150.4 140.1	0.5 0.4 0.8 1.9 2.1 5.2 6.4

### Emergency department visits

#### Cannabis-related poisonings

**Fig. 1:**
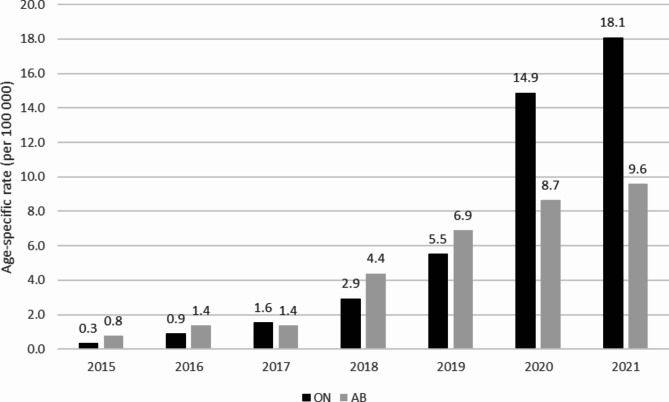
Total cannabis-related poisoning ED visit age-specific rates (per 100,000) for children 11 years or younger in Ontario and Alberta, from 2015 to 2021

Table 1; Fig. 1 depicts the annual age-specific rates of cannabis-related poisoning ED visits among children aged 11 years and younger for Ontario and Alberta from 2015 to 2021. For Ontario, from 2015 to 2019, there was a gradual increase in the age-specific rate of cannabis-related poisoning ED visits (from 0.3 to 100,000 to 5.5 per 100,000). From 2019 to 2020, the age-specific rate rose sharply to 14.9 per 100,000 and increased further to 18.1 per 100,000 in 2021. For Alberta, there was a gradual increase in cannabis-related poisoning ED visit age-specific rates from 2015 to 2021 (from 0.8 to 100,000 to 9.6 per 100,000). Based on the Joinpoint Regression trends analysis, pediatric cannabis-related poisoning ED visits for Ontario and Alberta increased significantly from 2015 to 2021 with an AAPC of 98.2% (95% CI: 79.1,119.2) and 57.4% (95% CI: 36.7, 81.2), respectively. The annual absolute change and the AAPC of the age-specific rates cannabis-related poisoning ED visits for Ontario and Alberta are presented in Table 2.

**Table 2:** Annual absolute change and average annual % change in pediatric cannabis-related poisoning ED visits in Ontario and Alberta, pediatric cannabis-related hospitalizations (Canada with the exception of Quebec) and pediatric edible-related cases, from 2015 to 2021

Outcome, Geographic coverage (*Data source*)	Cannabis-related poisoning ED visits Ontario *(NACRS)*		Cannabis-related poisoning ED visits Alberta *(NACRS)*		Cannabis edible-related poisoning cases ED visits *(CHIRPP)*		Cannabis-related poisoning hospitalizations Canada (except Quebec) *(DAD)*	
Year	Annual absolute change	Average annual % change (from Joinpoint)	Annual absolute change	Average annual % change (from Joinpoint)	Annual absolute change	Average annual % change (from Joinpoint)	Annual absolute change	Average annual % change (from Joinpoint)
2015	-	98.2% (95% CI: 79.1, 119.2)	-	57.4% (95% CI: 36.7, 81.2)	-	122.8% (95% CI: 64.0, 202.6)	-	63.4% (95% CI: 42.0, 87.9)
2016	0.6		0.6		N/A		-0.1	
2017	0.7		0.0		-0.1		0.4	
2018	1.3		3.0		19.1		1.1	
2019	2.6		2.5		19.4		0.2	
2020	9.4		1.8		107.3		3.1	
2021	3.2		0.9		-10.3		1.2	

#### Cannabis edible-related events (sentinel surveillance)

**Fig. 2:**
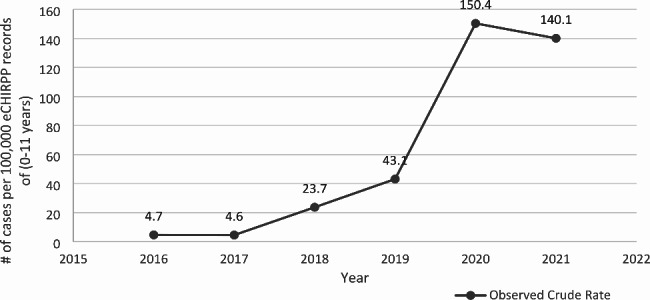
Annual distribution of cases associated with cannabis edibles, eCHIRPP, ages 0 to 11 years, per 100,000 eCHIRPP records in Canada

In total, 293 cannabis edible-related events were captured from 2016 to 2021 in CHIRPP. Figure 2 presents the overall trend of cannabis edible-related events among children aged 11 years and younger from 2016 to 2021. There was considerable variation in the rates during this period. The most substantial increase in age-specific rates was seen from 2019 to 2020. From 2016 to 2019, there was a steady increase in the age-specific rate of cannabis edible-related events (from 4.7 per 100,000 to 43.1 per 100,000 eCHIRPP records). From 2019 to 2020, the age-specific rate rose sharply to 150.4 per 100,000 eCHIRPP records and decreased slightly to 140.1 per 100,000 eCHIRPP records in 2021. Based on the Joinpoint Regression trends analysis, pediatric cannabis edible-related events increased significantly from 2016 to 2021 with an AAPC of 122.8% (95% CI: 64.0, 202.6). Annual absolute changes and AAPCs of the observed age-specific rates are presented in Table 2. The distribution of the types/forms of cannabis edible products captured in the injury/poisoning narrative description of the observed events is presented in Table 3. Almost half of all pediatric edible-related events involved gummy edible products (48.8%, n = 143), followed by edibles in the form of candy (12.6%), cookie (10.9%), and chocolate (8.9%). Based on the narrative description of the cannabis edible-related events, two themes emerged: 1) children inadvertently ingesting edible products due to access to such products/lack of safe storage practices (87.7% of cases), and 2) caregiver/family member unintentionally provided cannabis edible products to children (7.2% of cases).

**Table 3:** Frequency distribution of cases associated with cannabis edibles by type/form of edible product, CHIRPP, ages 0 to 11 years

*Type/Form of Edible Product*	Count	(%)
Gummy	143	48.8
Candy	37	12.6
Cookie	32	10.9
Chocolate	26	8.9
Brownie	8	2.7
Baked goods	9	3.1
Pills/Capsules	2	0.7
THC/OIL	1	0.3
Unspecified	35	11.8
***Total***	***293***	***100.0***

### Hospitalizations

**Fig. 3:**
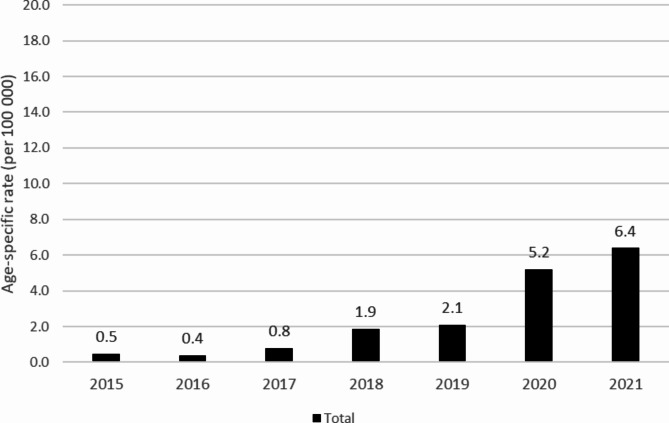
Total cannabis-related poisoning hospitalization age-specific rates (per 100,000) for children 11 years or younger in Canada (excluding Quebec), from 2015 to 2021

The annual age-specific rates of cannabis-related poisoning hospitalizations for Canada (excluding Quebec) are shown in Fig. 3. Similar to the pattern seen in Ontario ED visits from 2015 to 2019, there was a gradual increase in the age-specific rate of cannabis-related poisoning hospitalizations (from 0.5 to 100,000 to 2.1 per 100,000). From 2019 to 2021, the age-specific rate rose sharply to 6.4 per 100,000 in 2021. Based on the Joinpoint Regression trends analysis, pediatric cannabis-related poisoning hospitalizations increased significantly from 2015 to 2021 with an AAPC of 63.4% (95% CI: 42.0, 87.9). APC and AAPC of the age-specific cannabis-related poisoning hospitalization rates are presented in Table 2.

## Discussion

This study triangulated three data sources to better understand and capture the burden of pediatric cannabis poisonings across Canada. The age-specific rates of cannabis-related poisoning ED visits and hospitalizations of children between the ages of 0 to 11 increased significantly from 2015 to 2021. The largest increases in ED visits for the province of Ontario and hospitalizations for Canada (with the exception of Quebec) occurred from 2019 to 2020. Pediatric cannabis-related poisoning ED visits also increased significantly in Alberta from 2015 to 2021, but the most notable change was from 2017 to 2018, prior to the legalization of cannabis edibles. The largest observed increase in cannabis edible-related events, occurred between 2019 and 2020, coinciding with the largest increases in cannabis-related poisoning ED visits in Ontario, and in pediatric hospitalizations in Canada (with the exception of Quebec). Similarities in the patterns of the ED visits and sentinel surveillance system results suggest that some of the pediatric cannabis poisoning cases presenting to EDs and hospitals may be associated with the ingestion of edible cannabis products as opposed to other forms of cannabis. Overall, the pediatric cannabis poisoning ED and hospitalization rates were higher after legalization of cannabis (October 2018) and cannabis edibles (October 2019) compared to pre-legalization. This was consistent across all outcomes in this study. These findings provide additional support to existing evidence from the US [6–10] and from four provinces [11, 12, 14] in Canada.

It is important to note that poisonings with a milder clinical presentation (where the child presents with few or no obvious symptoms) may not be captured in hospitalization or ED visit data, as the child/caregiver may not seek formal care. However, poison centre data may reveal a greater burden of pediatric cannabis/edible poisonings as caregivers may reach out to poison centres for general information on managing symptoms at home and ask for guidance on whether they should seek formal care. In fact, data from the British Columbia Drug and Poison Information Centre show that the proportion of cannabis-related calls from 2013 to 2018 increased the most for those involving children between the ages of 0–12 compared to other age groups and that most of those calls were because the child had ingested cannabis edibles [17]. This highlights the importance of examining multiple data sources to get the most complete picture of cannabis-related poisonings in Canada. Future studies should consider reporting on poison centre calls, the type of cannabis involved and their origin to further assess the role that different cannabis products play in poisonings. Given that specific regulations on distribution and sale of cannabis can vary across Canadian jurisdictions, it could be important to explore this at the provincial and territorial levels.

The qualitative analysis from the sentinel surveillance data enriched our understanding of the findings by providing context surrounding edible-related cases among children. Close to nine in ten (87.7%) pediatric edible-related cases were due to lack of safe storage practices/easy access. This evidence supports previous recommendations that were made on the issue, particularly for regulations around safe storage and child-resistant packaging [7, 10, 12, 13, 17–19]. In addition, some of the pediatric cannabis edible-related poisonings (7.2%) occurred because a family member or caregiver was unaware that some of the sweets were edibles and accidentally gave them to children. These findings underscore the need to continue targeted public health interventions among adults related to (1) safe storage of cannabis products in households and (2) communication with family, friends, and caregivers related to cannabis edibles in the household to mitigate unintentional pediatric ingestion.

Another way to reduce cannabis-related harms among children would be to raise awareness on the risks associated with consuming illegal cannabis. Under the Cannabis Regulations, regulations pertaining to edibles, including restrictions on packaging (namely childproof and restrictions to mitigate appealing to youth) and quantity (capped at a maximum of 10 mg of THC per package), were created to reduce harms associated with consuming edible cannabis [2]. Illegal cannabis edible products are often not in childproof packaging and have colourful/appealing packaging, including look-alike packaging similar to recognizable confectionary products, and very high levels of THC, which can cause serious harm to children [2]. Some individuals may not be cognizant of the increased risk associated with illegal and unregulated cannabis products. The Government of Canada has published information on how to recognize legal and illegal cannabis, and why legal cannabis should be chosen [20]. Targeted public health messaging highlighting this resource could be beneficial. One systematic review [18] and one Canadian study [11] underlined the importance of determining whether legal or illegal products were more common among edible-related exposures among children. Unfortunately, most of the available Canadian data capturing cannabis-related harms are found in administrative data sets, which do not contain information on source of cannabis. Future studies are warranted to address this gap in evidence.

This is the first study reporting on trends in almost nationally representative rates of pediatric cannabis-related poisoning hospitalizations. This study addressed a gap in evidence flagged in two previous Canadian studies [11, 12] by providing novel data on trends for poisonings related to cannabis edibles captured through sentinel surveillance. Another key strength is the mixed-methods design of this study and the triangulation of three data sources, which provided additional context to the findings and helped better characterize public health and safety impacts of cannabis in Canada.

There are some limitations to note. First, the geographic coverage is incomplete. Cannabis-related poisoning hospitalization rates do not include Quebec, and age-specific rates of ED visits could only be derived for Ontario and Alberta. The CHIRPP collects data from select Canadian hospitals, and findings from the analysis may not represent all cannabis edible-related events in Canada. Second, the CHIRPP database is continuously updated and as a result, some years may not yet have complete data; however it is expected that the period covered in this manuscript will have a high completion rate as it does not include data from 2022. Third, there is some overlap between the datasets presented in this manuscript and supporting Canadian studies. We used the NACRS dataset for ED visits in Ontario and Alberta, which is the same dataset that was used in two existing Canadian studies [11, 12]. There is also some overlap with the CHIRPP dataset that was used in this study with a 2020 Canadian article that stated there were 35 edible cases from 2016 to 2019 [13]. Therefore the similarity in patterns of our results with those studies is not suggestive of a stronger trend. Fourth, Indigenous peoples including Inuit, Metis, and First Nations, and people who live in rural areas may be under-represented in the CHIRPP database. Fifth, lack of data on the source and type of cannabis ingested limits the analyses of cannabis poisoning ED visits and hospitalizations (not specific to edibles). While similarity in patterns of cannabis edible-related events from CHIRPP does seem to suggest that edible cannabis products may be contributing to the increasing rates in cannabis-related poisoning ED visits and hospitalizations, no definitive conclusion can be made. It should also be noted that significant events over time such as the 1) implementation of the Cannabis Act [1] in 2018, 2) amendment to the Cannabis Regulations [2] in 2019, and 3) the COVID-19 pandemic beginning in March 2020 may have influenced observed patterns and trends. As limited time has elapsed since cannabis legalization and regulation, caution is recommended when interpreting pre- and post-legalization trends based on data presented in this report. Sixth, health-seeking for substance-related harms may have changed due to public health measures implemented during the COVID-19 pandemic and may have led to an under-representation from March 2020 to June 2021. Analyses by CIHI show about 9,300 fewer ED visits per day across Canada compared to the pre-pandemic period (January to December 2019), with the greatest reduction among children and youth (0–19 years) [21]. The decrease in the rate of cannabis edible-related cases in 2021 compared to that in 2020 from CHIRPP records could also be attributed to the analysis by CIHI. Seventh, the rates presented in these analyses represent the most severe cannabis/edible-related poisoning cases, which is likely an under-representation of the true burden of pediatric cannabis poisonings in Canada. Lastly, it is possible that since cannabis has been legalized, parents and caregivers may feel more at ease disclosing the source of the poisoning (cannabis) and seek formal care for their child [11].

## Conclusion

Cannabis poisonings among children are a public health concern. In Canada, the age-specific rates of cannabis-related poisoning ED visits and hospitalizations among children 11 years and younger have increased between 2015 and 2021, with substantial increases being observed in hospitalizations across Canada (excluding Quebec) and ED visits in Ontario from 2019 to 2020. Similarly, the age-specific rate of cannabis edible-related cases from sentinel surveillance data also shows a similar increase. The triangulation of findings for all three data sources suggest that pediatric cannabis poisoning cases presenting to EDs and hospitals in Canada may be due to ingestion of edibles rather than other forms of cannabis. Results from this study underscore the importance of monitoring cannabis-related poisonings. To better understand the public health impact of cannabis-related harms, future studies could examine poison centre call data, as well as the source of cannabis and the potency of such products associated with poisonings among children. National health promotion strategies aiming at increasing public knowledge and risk-mitigation related to cannabis poisonings are essential. Public health messaging providing education on safe storage practices and labelling of cannabis products in households could help mitigate cannabis poisonings among children.

## Data Availability

The data that support the findings of this study are available from the Canadian Institute for Health Information (CIHI) and the Canadian Hospitals Injury Reporting and Prevention Program (CHIRPP) but restrictions apply to the availability of these data, which were used under license for the current study.

## Abbreviations

AAPC: Average Annual Percent Change
APC: Annual Percent Change
CHIRPP: Canadian Hospitals Injury Reporting and Prevention Program
CI: Confidence Interval
CIHI: Canadian Institute for Health Information
DAD: Discharge Abstract Database
ED: Emergency Department
ICD: International Statistical Classification of Diseases
IRR: Incident Rate Ratio
NACRS: National Ambulatory Care Reporting System
PHAC: Public Health Agency of Canada
US: United States
